# Advanced spot quality analysis in two-colour microarray experiments

**DOI:** 10.1186/1756-0500-1-80

**Published:** 2008-09-17

**Authors:** Mikalai Yatskou, Eugene Novikov, Guillaume Vetter, Arnaud Muller, Emmanuel Barillot, Laurent Vallar, Evelyne Friederich

**Affiliations:** 1 Microarray Center/LBMAGM, CRP-Santé, 84 Rue Val Fleuri, L-1526, Luxembourg; 2 Cytoskeleton and Cell Plasticity Laboratory, Life Sciences Research Unit, University of Luxembourg, 162A Avenue de la Faïencerie, L-1511, Luxembourg; 3 Institut Curie, Service Bioinformatique, Institute Curie, 26 Rue d'Ulm, Paris, F-75248, France; 4 INSERM, U900, Paris, F-75248, France; 5 Ecole des Mines de Paris, Fontainebleau, F-77300, France

## Abstract

**Background:**

Image analysis of microarrays and, in particular, spot quantification and spot quality control, is one of the most important steps in statistical analysis of microarray data. Recent methods of spot quality control are still in early age of development, often leading to underestimation of true positive microarray features and, consequently, to loss of important biological information. Therefore, improving and standardizing the statistical approaches of spot quality control are essential to facilitate the overall analysis of microarray data and subsequent extraction of biological information.

**Findings:**

We evaluated the performance of two image analysis packages MAIA and GenePix (GP) using two complementary experimental approaches with a focus on the statistical analysis of spot quality factors. First, we developed control microarrays with a priori known fluorescence ratios to verify the accuracy and precision of the ratio estimation of signal intensities. Next, we developed advanced semi-automatic protocols of spot quality evaluation in MAIA and GP and compared their performance with available facilities of spot quantitative filtering in GP. We evaluated these algorithms for standardised spot quality analysis in a whole-genome microarray experiment assessing well-characterised transcriptional modifications induced by the transcription regulator SNAI1. Using a set of RT-PCR or qRT-PCR validated microarray data, we found that the semi-automatic protocol of spot quality control we developed with MAIA allowed recovering approximately 13% more spots and 38% more differentially expressed genes (at FDR = 5%) than GP with default spot filtering conditions.

**Conclusion:**

Careful control of spot quality characteristics with advanced spot quality evaluation can significantly increase the amount of confident and accurate data resulting in more meaningful biological conclusions.

## Background

Microarray technology allows gaining novel insights into different biological phenotypes by studying genome-wide differences in gene expression profiles [1,2]. Many efforts have been made to standardize microarray data analysis pipelines [3,4]. Several initiatives such as the MAQC project showed that standardising data analysis procedures improved performance of microarray platforms [5]. A critical component of the microarray data analysis pipeline is image analysis. Any error made at this stage of the analysis may propagate throughout the pipeline invalidating final biological conclusions such as differential expression or gene network establishment. Among the various approaches aiming at improving microarray analysis, one of the most important and less formalized is the evaluation of the quality of spots obtained in microarray experiments [6]. Too stringent spot quality requirements can result in filtering-out relevant spots and loss of useful biological information. Conversely, too flexible filtering conditions will conserve bad spots leading to wrong predictions. This situation is mainly observed when analysing weak or contaminated spots which yet might contain important biological information. Numerous studies are aimed at improving the control of microarray spot quality including spot quality assessment [7] and filtering [8], evaluation of normalisation procedures [9], missing values imputation [10], comparison of different spot quality-assessing algorithms [11]. However, there is still a lack of consistent and standardized methodology for microarray image analysis using advanced algorithms for automated spot quality evaluation.

Several software tools, such as AMIA [12], Matarray [13], MASQOT-GUI [14], Tiger Spotfinder [15], MAIA [16], which are based on standardized, semi-automated strategies for microarray image analysis are currently available for academic users. GenePix Pro (Molecular Devices, Sunnyvale, CA, USA) is a representative commercial software that is routinely used. Among reported software [for a brief overview see Additional file 1] GenePix (GP) and MAIA have distinct advantages. GP provides automated and user-friendly tools for microarray gridding, feature alignments, data management, and graphical representation of the results. GP has also functionality for spot quality analysis, in particular, a filter system for flagging spots as "good" (Flag = 100) or "bad" (Flag = 0) based on a user-defined set of conditions for the GP parameters. However, this facility is not automated and spot qualification in GP is highly dependent on user decisions. We have chosen MAIA as a representative example of an automated spot quality treatment allowing to save spots containing useful biological information [16]. MAIA implements a compact set of statistical algorithms for microarray image analysis, including algorithms for the spot quality analysis at the pixel level. MAIA assigns to each ratio estimate a quality score ranging from 0 to 1. This score is calculated from 10 main quality characteristics reflecting different spot properties within the microarray.

Here, we developed advanced spot quality evaluation methodologies for MAIA and GP. These approaches were evaluated experimentally and compared to the default parameter filtering settings provided in GP. The precision and accuracy of spot quantification procedures were verified using microarrays with *a priori *known ratios of Cy5 to Cy3 intensities and biological relevance was assessed by comparing differentially expressed genes and significantly over-represented gene ontology (GO) categories in a whole-genome transcriptomic microarray experiment. Our results show that advanced spot quality evaluation methodologies developed in MAIA give slightly more accurate and precise Log_2 _ratios of signal intensities allowing to recover more useful spots and differentially expressed genes when compared with the default spot filtering procedure in GP.

### Methodology for spot quality evaluation

#### Semi-automatic pipeline in MAIA

**Figure 1:**
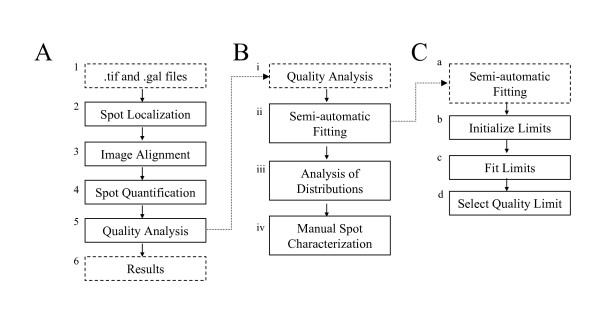
**The workflow diagram of the semi-automatic analysis pipeline in MAIA**. (A) Main scheme of the semi-automatic analysis pipeline in MAIA. (B) Scheme of the spot quality analysis using the quality parameters. (C) Scheme of the semi-automatic fitting of the parameter limits.

The main principles of the semi-automatic image analysis in MAIA are briefly outlined [6]. The general image processing workflow is shown in Figure 1A. In Block 1, raw data, i.e. .*tif *and .*gal *(GenePix Array List) files, are imported in the program, whereupon the automatic image analysis procedure is launched: *Spot Localization *(Block 2), *Image Alignment *(Block 3), *Spot Quantification *(Block 4) and *Quality Analysis *(Block 5). Part of the procedure presented in Figure 1A, Blocks 1–4, is also applicable to GP. To standardize procedures for spot quality evaluation in MAIA, we expand the block *Quality Analysis *(Block 5) into three main steps, presented in Figure 1B: semi-automatic fitting of the quality parameter weights (Block ii), analysis of the histograms/distributions of the quality parameters (Block iii); and manual spot characterization (Block iv). Semi-automatic fitting (Figure 1C) of the quality parameter weights can only be launched for an image comprising replicated spots [6]. In Block b the initialization of the quality parameter limits is performed. The quality parameter weights are then fitted in Block c *Fit Limits*, yielding appropriate parameter limits for quality assessment. In Block d a proper value of the *Quality Limit *is defined to reach an acceptable statistical error range. The *Fit limits *procedure from Block c may over- or under-estimate limits of the quality parameters. Identifying a sub-set of relevant quality parameters can be achieved by analyzing their distributions (Block iii, Figure 1B), a step which also allows refining the limits of the quality parameters.

#### Spot filtering in GP

Many microarray studies performed with GP use standard or default settings of GP criteria and parameter limits for filtering spot features. An example of the use of default parameters in GP, named here *standard *parameters, is shown in Table 1[17]. However, the standard set of GP parameters is not always optimal and may result in losing many informative spots. To systematize GP filtering, we tested various sets of the GP parameters and their cutoff values to define a set of parameters preserving a maximum of informative spots. We analysed 56 parameters provided by GP and classified them into nine groups, representing logically-formalized properties of a spot in a microarray [see Additional file 1]. These groups were established using hierarchical clustering with the Pearson centered metrics applied to a representative microarray. By combining proper parameter representatives of each correlation group with spot properties, we reduced the full set of the GP parameters to a limited number of the most representative parameters which were further used for automatic filtering in GP. From the distributions, we defined three groups of filtering conditions for detecting "good" spots associated with three levels of filtering stringency (*weak*, *medium *and *strong/stringent*) and corresponding to 1, 2, and 3 STD borders of the tails in the parameter distributions. List of selected GP parameters and summary of the estimated parametric constrains are shown in Table 1. Additionally, so-called *standard *configuration of default GP parameters and filtering limits provided in the GP and Acuity software [17] are given in Table 1 and are used in the following analysis.

**Table 1:** The GP parameters and limits for different filtering conditions in GP.

		**Filtering conditions**			
	**Parameter**	**Strong**	**Medium**	**Weak **	**Standard**

1	*Dia.*	>= 120	>= 100	>= 70	NR
2	*% > B635+2SD*	> 98	> 95	> 66	> 55*
3	*% > B532+2SD*	> 98	> 95	> 66	> 55
4	*F635% Sat.*	< 1	< 5	< 10	< 2–3
5	*F532% Sat.*	< 1	< 5	< 10	< 2–3
6	*Rgn Ratio (635/532)*	> 0	> 0	> 0	NR
7	*Rgn R2 (635/532)*	> 0.8	> 0.6	> 0.4	> 0.5
8	*Circularity*	> 85	> 80	> 50	> 80
9	*F635 Median – B635*	> 600	> 400	> 200	NR
10	*F532 Median – B532*	> 600	> 400	> 200	NR
11	*SNR 635*	> 10	> 5	> 2	> 3
12	*SNR 532*	> 10	> 5	> 2	> 3
13	*Flags*	>= 0	>= 0	>= 0	>= 0

The table summarises the main parameters and limits for different filtering conditions used in GP. Other parameters and limits that are used in default/standard filtering setting of GP/Acuity are: [*Sum of Medians (635/532)*] > 200–500, [*B532 CV*] < 25, [*B635 CV*] < 25, LCase([*ID*]) <> "empty". NR indicates that a corresponding parameter is not a default parameter as recommended in GP.

#### Microarray analysis pipeline

A brief overview of steps common to MAIA and GP of our microarray analysis pipeline (normalization, preprocessing, aso) are given below.

**Figure 2:**
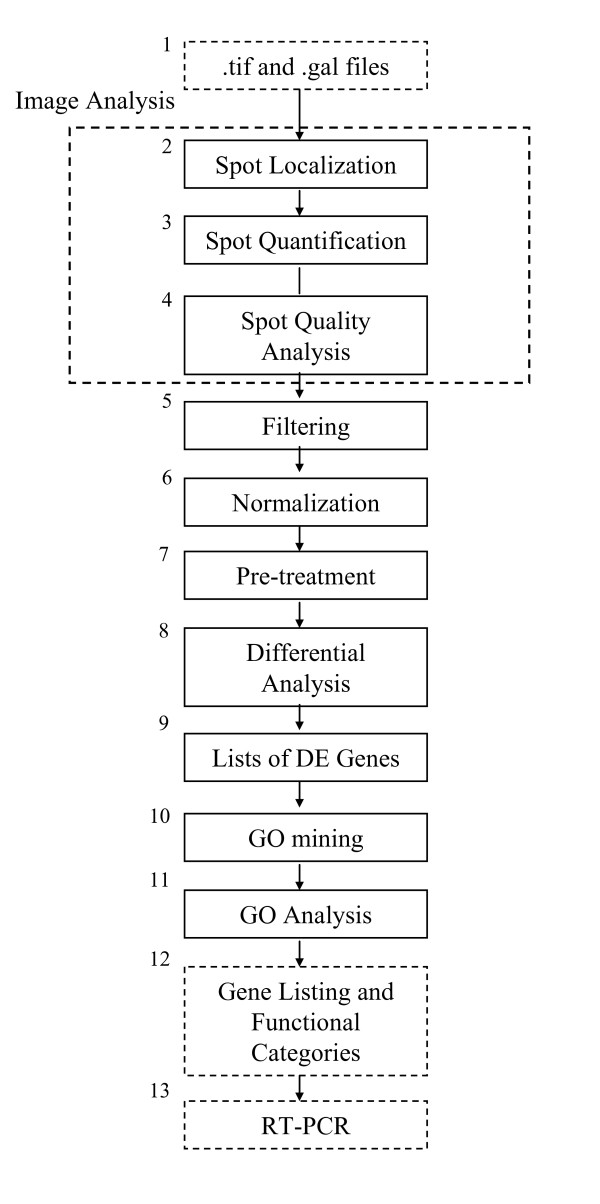
Overall flow diagram of the statistical analysis of microarray data.

The overall flow of statistical analysis of microarray data performed in this study is shown in Figure 2. Data processing starts with import of the scanned microarray image files (.*tif *files) and GenePix Array List file (.*gal *file), in Block 1. Block 2–4 represents microarray image analysis that can be performed either by MAIA or GP. *Image Analysis *consists of *Spot Localization *(Block 2), *Spot Quantification *(Block 3) and *Spot Quality Analysis *(Block 4). For Spot Quality Analysis, the spots are further quantified using several statistical quality factors, which are also used in subsequent filtering (Block 5). The microarray data are normalized (Block 6), and then series of microarray data are combined in one dataset table (Block 7) for preliminary treatment, which includes procedures of dye-swap transformation, evaluation and correction of genes with missing values, data centring and scaling, data visualisations (box plots, histograms of the Log_2 _ratios, MA-plots). Differential analysis of gene expression data from replicated microarrays is done (Block 8), differentially expressed genes at the accepted false-discovery rate are identified (Block 9) and then submitted for GO mining (Block 10). The results of GO mining are analyzed (Block 11) to identify the relative enrichment of significant functional categories. The data analysis pipeline yields lists of differentially expressed genes and significantly enriched biological categories (Block 12). Microarray data are validated using the RT-PCR or qRT-PCR techniques applied to a set of selected genes (Block 13).

### Experimental evaluation of standardized approaches for spot quality assessment using control microarrays with a priori known fluorescence ratios

**Figure 3:**
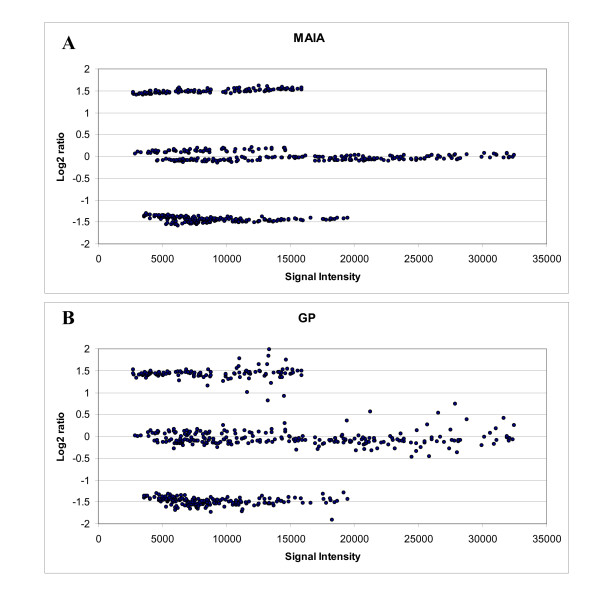
**Log_2 _ratio vs Signal Intensity scatter plots obtained by MAIA (A) and GP (B) for a selected control microarray**. The fixed number of *down*-, *invariant*-, and *up*- features, found "good" by both programs, is selected for the statistical comparison. Procedures of girding and image quantifications were done automatically as recommended in the corresponding manuals of MAIA and GP. A control microarray consists of three populations of spots with expected Log_2 _ratios of -1.58 (3:1), 0 (1:1), and 1.58 (1:3) [see Additional file 2]. Data yielded by the GP analysis are more dispersive. We assume that no normalisation procedure is required for these data even at low signal intensities, where the experimental data may deviate from the theoretical values. Data are not calibrated and not normalized.

Spot quality can be evaluated in terms of i) accuracy and precision of the obtained ratio estimates and ii) quantitative characterization of various spot defects or systematic distortions, such as dust, donut-shaped spots, smears, outliers, saturated and high-background pixels, non-linear foreground and background signals. First, we evaluated the accuracy and precision of the algorithms for ratio estimation. For this purpose, we developed five dedicated microarrays referred as to control chips using the *Arabidopsis thaliana *spike RNA control set produced by the Institute for Genomic Research [18]. The control microarrays actually consist of three populations of spots further noted as *down*-, *invariant*-, and *up*- features [see Additional file 2]. Spots deemed as relevant by both MAIA and GP in all five microarray replicates were selected and the corresponding means of signal intensity Log_2 _ratio were analysed. First, we directly compared non-normalized data of *down*- and *up*-features immediately after spot quantification. Then, we compared these data upon calibration to remove any linear bias introduced by the quantification algorithms. This transformation consisted in a linear normalization of the Log_2 _ratios so that the set of *invariant *features, i.e. features with 1:1 theoretical ratios, was centred. A brief summary of comparison of 5 control slides is given in Table 2 (for a full version of Table 2 see Additional file 3). The plots *Log*_2 _*Ratio vs Intensity *of a typical control microarray obtained in MAIA (top) and GP (bottom) are shown in Figure 3. Three groups of spots were found with mean Log_2 _ratios corresponding to those expected, but being more dispersed when analysed using GP. We also noted that MAIA gives more precise estimations of the Log_2 _ratios. Analysis of the relative errors showed that estimations of the Log_2 _ratios by MAIA were on average 5–7% closer to the expected ones as compared to those found with GP. After calibration the difference became less significant. A paired t-test of two samples assuming equal variances also showed that the differences in means were significant for the non-calibrated data. For the calibrated data, subtle differences were observed only for *dow*n-features. Discrepancy in the expression ratios may be due to different quantification strategies in the two programs. Indeed, the GP manual recommends using medians to estimate the foreground and background fluorescence intensities in Cy3 and Cy5 colour channels to create the final ratio estimate. In MAIA, the mean estimates are used instead. Although the median estimates ensure more robust ratio values, it is known that they have larger standard deviations than means [19]. As in MAIA the mean estimates are calculated after special outlier-filtering procedure [20], these estimates are robust and better preserve precision of the final ratios.

**Table 2:** Mean Log_2_ratio obtained from analysis of five control slides by MAIA and GP.

		**Log_2 _ratio**		**Calibrated Log_2 _ratio**	
**Slide**	**Population**	**MAIA**	**GP**	**MAIA**	**GP**

**Slide 1**	*Down*	-1.74(0.10)	-1.83(0.11)	-1.47	-1.45
	*Up*	1.26(0.08)	1.14(0.18)	1.53	1.52
**Slide 2 **	*Down*	-2.07(0.11)	-2.19(0.13)	-1.46	-1.45
	*Up*	0.92(0.10)	0.79(0.13)	1.53	1.54
**Slide 3**	*Down*	-1.33(0.13)	-1.44(0.15)	-1.58	-1.55
	*Up*	1.75(0.10)	1.60(0.13)	1.50	1.50
**Slide 4**	*Down*	-1.55(0.15)	-1.68(0.18)	-1.56	-1.54
	*Up*	1.53(0.12)	1.35(0.17)	1.51	1.49
**Slide 5**	*Down*	-1.44(0.06)	-1.48(0.12)	-1.43	-1.44
	*Up*	1.49(0.04)	1.45(0.19)	1.50	1.49

The mean Log_2 _ratio obtained from analysis of five control slides by MAIA and GP for *down*- and *up*- notated populations of features with expected Log_2 _ratios of -1.58 and 1.58. The standard variation of the mean Log_2 _ratio is shown in brackets. The p-values from the paired t-test for the Log_2 _ratios are less than 1.4E-3. The p-values from paired t-test for the calibrated Log_2 _ratios are in range [1.7E0-7; 0.72]. For the full version of Table 2, including p-values from the paired t-test and estimated errors for the Log_2 _ratio, see Additional file 3.

Collectively, our data show that MAIA generally gives slightly more accurate and precise Log_2 _ratios. An additional processing step or a proper calibration of the data is therefore needed in GP to smooth out the differences in Log_2 _ratios between the programs. This should allow conserving more informative spots in the follow-up analysis.

### Evaluation of the performances of the image analysis methodologies in a comparative gene expression study using whole-genome arrays

Although accuracy and precision of the ratio estimates are important for reliable follow-up analysis, the major problem in microarray studies comes from deficient spots that when being improperly treated, may obscure the final conclusions. However, while stringent filtering conditions allow eliminating such bad spots, they also might lead to the loss of good, informative spots.

Our artificial microarrays with the known ratios are of very good overall quality and therefore they are not appropriate to evaluate algorithms for quantitative characterization of various spot deficiencies or systematic distortions. To evaluate the developed filtering procedures, we used oligonucleotide microarrays measuring genes which are differentially expressed in human MCF-7 epithelial breast carcinoma cells after induction of the transcription regulator SNAI1 [see Additional file 2]. SNAI1 directly represses the expression of a set of genes triggering thereby a well-described transcriptomic program which leads to the transition of epithelial cells to a mesenchymal phenotype [21]. Because functional categories of genes that are up- or down-regulated during this process are well-characterized [22], we considered this experimental model suitable to further evaluate the performance of the image analysis procedures. We used cells transfected with the human SNAI1-cDNA cloned in a tetOff conditional expression system. Expression profiles before and after SNAI1 induction (time points 0 and 96 hours, a sample at time point 0 was a reference) were analyzed using oligonucleotide two-color microarrays purchased from the "University Medical Center of Utrecht" (UMCU, The Netherlands) [23]. The microarray images were analysed either by MAIA or GP as described in Figure 2. We applied the semi-automatic approach for the spot quality assessment in MAIA and four filtering conditions – *standard, weak*,*medium*, and *strong *(defined in the Table 1) – for the spot quality assessment in GP. For the analysis in GP, we arbitrarily selected one microarray out of the 9 in the series and performed automatic gridding and spot quantification procedures. *Weak*, *medium *and *strong/stringent *filtering conditions were defined as described in Additional file 1 by considering 1, 2 and 3 STD borders in the distributions of the GP parameters (see in Table 1). We used the default GP filtering parameters as *standard*.

**Figure 4:**
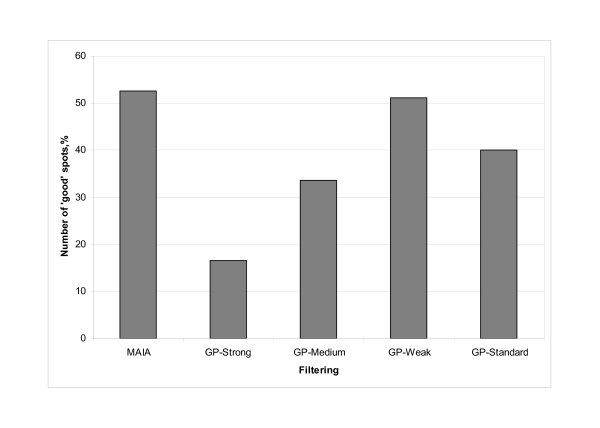
**Average number of "good" spots obtained from spot quality analysis of the whole-genome microarrays using MAIA and four filtering approaches in GP**. Series of nine whole-genome microarrays, slides containing 25 392 spots (21 521 70-mers and 3 871 control spots, Operon, human whole genome version 2.0), were printed onto Corning UltraGAPS slides with 48 subgrids of 23 × 23 spots. Differential expression of genes after SNAI1 induction was analysed. Data were analysed using the semi-automatic approach for the spot quality assessment in MAIA and four filtering conditions – *standard, weak*, *medium*, and *strong *(defined in the Table 1) – for the spot quality assessment in GP resulting in two groups of spots: "good" and "bad". The bars show the average number of "good" spots, calculated as a mean of nine microarrays, obtained from spot quality analysis either by MAIA or GP. Results are expressed as percentages relative to the total number of spots in each microarray.

The average number of spots deemed as "good" using MAIA and GP programs, calculated as a percentage of total number of spots in a microarray (25 392), is plotted in Figure 4. MAIA detected 13%, 19%, and 36% more "good" spots than GP with *standard*, *medium *and *strong *filtering conditions, respectively. Moreover, the number of good-quality spots found with MAIA, 53%, was comparable to that obtained by the GP-weak filtering, 51%. Obviously, the proportion of "good"-quality spots might be increased technically by releasing filtering conditions defined by cutoff values for the GP parameters. This would lead to an increase of the number of true "good" spots (true positives), but would also generate more false "good" spots (false positives). We found that a majority of the spots identified as "good" in MAIA but not in GP, independent of the stringency of the filtering procedure in GP, were associated with saturated and/or contaminated pixels or with low signals or high background levels [see Additional file 4]. To address the biological relevance of these extra "good" spots found in MAIA compared to GP, we further analyzed the differentially expressed (DE) genes. To determine their significance level and their biological value we used the SAM and GoMiner programs, respectively [24,25]. The SAM (Significance Analysis of Microarrays) is a modified t-test for finding significantly expressed genes in a set of microarray experiments. GoMiner, a program package, organizes lists of DE genes from a microarray experiment for gene ontology-based biological interpretation. This analysis provides quantitative and statistical output on enrichment or depletion of bio-categories of DE genes.

**Figure 5:**
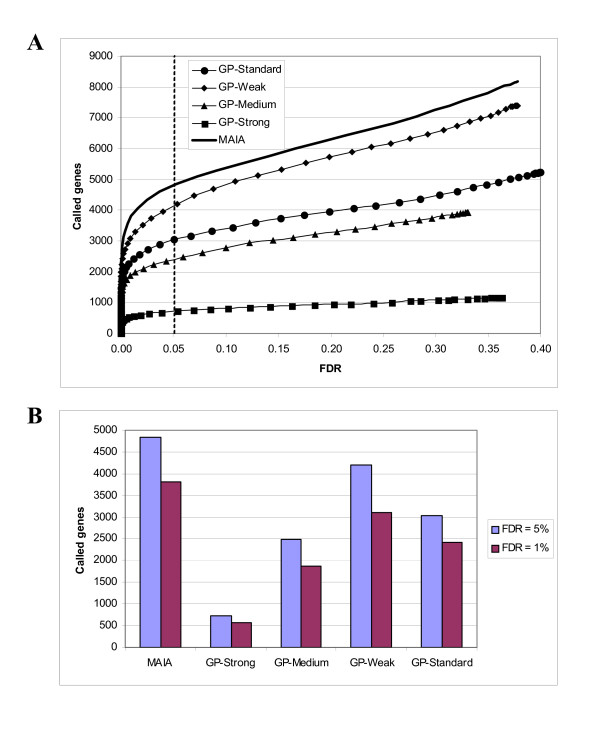
**The SAM plots of the nine whole-genome microarrays resulted from spot quality analysis in MAIA and GP**. (A) Number of called genes [24] versus the false discovery rate (FDR) as resulted after SAM analysis of gene lists generated by MAIA and GP-associated analysis. (B) The number of differentially expressed genes (called genes) at FDR = 5 and 1% found by SAM.

To identify the DE genes from the "good" spot datasets produced by MAIA and GP we applied one-class response type of SAM. Summary of the SAM plots resulting from the analysis of each (MAIA or GP) "good" spot dataset is shown in Figure 5. At the cutoff FDR = 5% the MAIA spot quality analysis preserved 12% more significant genes than GP *weak *filtering conditions, 38% and 47% more significant genes than GP *standard *and *medium *filtering conditions and even more (85%) with more stringent GP filtering conditions. The number of DE genes found after *medium *and *standard *filtering was in a close range, remaining at the same proportion as for deemed "good" spots. However, *medium *and *standard *filtering yielded about 38–47% less DE genes than those obtained by analysis in MAIA. In addition the GP *weak *filtering did not yield as many DE genes when compared with MAIA, albeit the input was similar, 51 and 53%, respectively. Nevertheless, the list of the GP *weak *DE genes exceeded over those obtained from the GP *standard *and *medium *filtering conditions. This is additional proof that the default filtering parameters in GP may be sub-optimal and need to be improved and to be automated. The GP *strong *filtering gave the shortest list of DE, corresponding to 20% of the MAIA DE gene list, and to highly-expressed genes in the study, i.e. genes associated with high-quality spots in the arrays.

Significant DE genes identified by SAM analysis (FDR cutoff 5%) of all "good"-quality spots in MAIA and GP were submitted to GoMiner to identify the over-represented GO categories as compared to the overall GO categories represented on the whole microarray. GO analysis highlighted a marked enrichment in GO categories in the gene dataset obtained from MAIA. Indeed, 278 enriched GO categories were identified in the dataset from MAIA, compared with 179 from GP-*standard*, 202 from GP-*weak*, 204 from GP-*medium*, and 112 from GP-*strong *(p-value cutoff 5%, FDR cutoff 30%) [see Additional file 5]. Interestingly, the GP-*weak *filtering did not introduce much more biological terms than the GP-*standard *filtering, indicating that lowering the spot filtering conditions in GP did not improve gene data mining or statistical significance of the GO categories. Analysis of the GO terms after the GP-*standard *filtering revealed that the majority of functional categories were similar to those obtained by MAIA although leading to higher p-values and FDR scores [see Additional file 5]. When considering GO categories potentially playing an important role in SNAI1 activity and EMT process, p-values of the Fisher exact test resulting from the GoMiner analysis were slightly lower with MAIA dataset than those obtained with GP (Table 3). Among the GO categories listed in Table 3, some were most important bio-functions involved in the EMT processes. For example, the categories *regulation of cell cycle *and *cell growth *indicated a direct blocking of these functions, after the ectopic expression of the *SNAI1 *protein [26]. *Wnt*-pathway is known to contribute to the EMT [27] and *Vitamin D receptor *has been shown recently to be directly regulated by the *SNAI1 *protein [28]. Finally, ectopic expression of SNAI1 target genes is involved in the establishment of cytoskeleton organization [29]. Our results suggested that the larger number of significant DE genes obtained in MAIA is related to the net increase in enriched GO categories. This could be very helpful when analysing the contribution of groups of genes in specific biological processes.

**Table 3:** p-values of selected GO categories resulted from the different conditions of analysis.

**GO term**	**MAIA**	**GP-standard**	**GP-weak**	**GP-medium**	**GP-strong**
*Kinase activity*	0.000	0.002	0.000	0.088	0.849
*Regulation of cell cycle*	0.001	0.039	0.014	0.013	0.201
*Hormone receptor binding*	0.006	0.006	0.073	0.093	0.307
*Wnt receptor activity*	0.016	0.732	0.053	0.258	1.000
*Actin cytoskeleton*	0.063	0.088	0.134	0.164	0.102
*Transcriptional repressor activity*	0.003	0.007	0.000	0.017	0.029
*Transcriptional activator activity*	0.026	0.352	0.177	0.685	0.439
*Vitamin D receptor binding*	0.005	0.003	0.013	0.041	1.000
*Regulation of cell growth*	0.066	0.094	0.016	0.022	0.868

Several biological categories with a well-established role in epithelial to mesenchymal transition (EMT) were selected for comparison in terms of the p-values. p-values obtained for the GP-*strong *gene list have the weakest statistical meaning. Statistical results yielded from GP-*standard*, *weak*, and *medium *filtering are in mutual agreement. MAIA gave the best p-values among all tested spot quality approaches. Presented categories were obtained with FDR < 0.3.

To compare various spot quality evaluation approaches using MAIA and GP, we established a list of *bone fide *DE genes. To do so, we first randomly selected about a hundred genes based on their differential expression behaviour and their confirmed or potential function in EMT, as supported by literature search. This approach enabled us enriching the list for "true" DE genes. Out of this first list, we randomly selected 24 genes the differential expression of which was confirmed by the RT-PCR and qRT-PCR (Table 4). Then, we determined how many of these 24 confirmed genes were detected by various spot quality evaluation approaches. Summary of this comparison, including genes annotation and description, is presented in Table 4. 14 genes were detected using the GP-*standard *conditions, 17, 9, 2 were included in the GP-*weak*, GP-*medium*, GP-*strong *lists, respectively. All 24 genes were found in the MAIA DE gene list.

**Table 4:** Comparison of MAIA and GP spot filtering approaches on a set of 24 selected genes confirmed by RT-PCR or qRT-PCR.

**Symbol**	**Description**	**MAIA**	**GP-standard**	**GP-weak**	**GP-medium**	**GP-strong**
	*Down*					
*KLF5*	Krueppel-like factor 5	+	+	+	+	
*TJP3*	Tight junction protein ZO-3	+	+	+		
*KRT12*	Keratin, type I cytoskeletal 12	+	+	+		
*BSPRY*	B-box and SPRY domain containing	+		+		
*CORO1A*	Coronin-1A	+	+	+	+	
*STAP2_HUMAN*	Signal-transducing adaptor protein 2	+		+		
*PPP1R16A*	Protein phosphatase 1 regulatory subunit 16A	+	+	+	+	+
*KRT18*	Keratin, type I cytoskeletal 18	+				
*STMN3*	Stathmin-3	+	+	+	+	
*TRIB3*	Tribbles homolog 3	+		+		
*CLDN3*	Claudin-3	+				
*TXNIP*	Thioredoxin interacting protein	+				
						
	*Up*					
*MSX1*	Homeobox protein MSX-1	+	+	+	+	
*GULP1*	GULP, engulfment adaptor PTB domain containing 1	+	+	+	+	
*DUSP2*	Dual specificity protein phosphatase 2	+	+	+	+	
*ID3*	DNA-binding protein inhibitor ID-3	+				
*THBD*	Thrombomodulin precursor	+	+	+		
*HS6ST2*	Heparan sulfate 6-O-sulfotransferase 2	+	+	+		
*TGFBI*	Transforming growth factor-beta-induced protein ig-h3 precursor	+				
*S*100*A*10	Calpactin I light chain	+				
*SERPINH1*	Collagen-binding protein 2 precursor	+				
*SNAI2*	Zinc finger protein SLUG	+	+	+		
*COL5A1*	Collagen alpha 1(V) chain precursor	+	+	+	+	
*ANXA2*	Annexin A2	+	+	+	+	+

RT-PCR and qRT-PCR analysis of 24 selected genes was performed to validate microarray data. Then, the presence of these genes in lists of DE genes obtained in the whole-genome microarray experiment was determined using different spot quality evaluation approaches in MAIA and GP. Fourteen genes were detected using the GP-*standard *conditions, 17, 9, 2 were present in the GP-*weak*, GP-*medium*, GP-*strong *lists, correspondingly. All 24 genes were found in the MAIA DE gene list. The sign "+" indicates if a gene is present in the corresponding DE gene list.

## Conclusion

Altogether, our data indicate that MAIA is a robust microarray image analysis program allowing a more accurate spot quantification and an improved collection of significant and relevant DE genes compared to GP. When considering GO categories potentially playing an important role in SNAI1 activity and EMT process, statistically enriched categories obtained by a GoMiner analysis had slightly lower p-values with MAIA dataset than those obtained with GP. Due to a larger number of significant DE genes, MAIA ensures a net increase in enriched GO categories. This could be very helpful when looking for subtle contribution of some biological processes. More generally, this study showed that careful control of spot quality characteristics with advanced spot quality evaluation can significantly increase the amount of meaningful data yielding more confident and accurate biological conclusions.

## Competing interests

The authors declare that they have no competing interests.

## Authors' contributions

MY developed the advanced spot quality evaluation procedures in GP and MAIA and drafted the manuscript. EN assisted in improving spot quality analysis in MAIA and drafted the manuscript. GV carried out the whole-genome microarray experiment, RT-PCR and qRT-RTP validation experiments. AM conducted the gene annotation mining and analysis. LV developed the control microarrays, contributed to gene-expression experiments, and drafted the manuscript. EF, LV, and EB conceived the study, participated in its design and coordination. All authors read and approved the final manuscript.

## Supplementary Material

Additional file 1 Methodology for spot quality evaluation. Technical details of the image analysis procedures using semi-automatic pipeline in MAIA and analysis of the parameters and spot filtering in GP. A brief overview of freeware image analysis software is giving. Click here for file

Additional file 2 Methods. Microarray fabrication, preparation of spiked RNA samples, microarray experiments, establishment of MCF-7 cell lines conditionally expressing SNAI1, RT-PCR and quantitative RT-PCR (qRT-PCR), statistical analysis of microarray data, software. Click here for file

Additional file 3 Summary of results obtained from the analysis of five control slides by MAIA and GP. The full version of Table 2 including p-values from the paired t-test and estimated errors for the Log_2 _ratio. Click here for file

Additional file 4 Informative spots in the whole-genome microarray. Figure shows the images (left) and the Cy5/Cy3 scatter plots (right) of two typical informative spots that were removed by any filtering in GP and saved in MAIA during analysis of the whole-genome microarray data obtained in the SNAI1 induction experiment. (A) – A spot affected by a relatively high saturation effect, (B) – A spot affected by a high background intensity effect. Click here for file

Additional file 5 Results of gene ontology analysis. Results of bio-categories and statistical scores obtained from the gene ontology analysis. Click here for file
