# Supplementary material for: Advanced spot quality analysis in two-colour microarray experiments
Source: BMC Res Notes. 2008 Sep 17;1:80. doi: 10.1186/1756-0500-1-80 (PMC2556690; doi:10.1186/1756-0500-1-80)
Supplement: Additional file 1 — Methodology for spot quality evaluation. Technical details of the image analysis procedures using semi-automatic pipeline in MAIA and analysis of the parameters and spot filtering in GP. A brief overview of freeware image analysis software is giving. [file 1756-0500-1-80-S1.pdf]

Methodology for spot quality evaluation

Semi-automatic pipeline in MAIA

The general workflow of the semi-automatic pipeline analysis in MAIA is shown in Figure 1A, Manuscript. In Block 1 raw data, i.e. *.tif* and *.gal* (GenePix Array List) files, are imported in the program. The automatic image analysis consists of *Spot Localization* (Block 2), *Image Alignment* (Block 3), *Spot Quantification* (Block 4) and *Quality Analysis* (Block 5). We refer to [1] and [2] for detailed description of the algorithms for spot localization and spot quantification implemented in GP and MAIA, respectively.

To advance procedures for evaluation of the spot quality (Block 5), we describe the corresponding filtering procedures in more detail. Besides the ratio estimates, the spot quantification procedure (Block 4) generates a table with ten quality parameters, characterizing different features of the spots. These characteristics are scaled to the unique quality bar in a range between 0 and 1, and the integral, overall spot quality score, is estimated. The *Quality Analysis* (Block 5) consists in three main steps, presented in Figure 1B: semi-automatic fitting of the quality parameter weights (Block ii), analysis of the histograms/distributions of the quality parameters (Block iii); and manual spot characterization (Block iv).

Semi-automatic fitting of the quality parameter weights can be launched only for an image having replicated spots. The results of *Spot Quantification* are displayed in the *Quality Plot* (Figure S1 D). In this plot a dot represents a replicate with *Overall Quality* value at *y*-axis and *Ratio CV* at *x*-axis. *Overall Quality* is defined as

$$Q_k = \min_j \{ \min_i \{ q_{kji}^{w_i} \} \} \qquad (1)$$

where q_{kji} is the i-th quality parameter of the j-th replicated spot in the k-th replicate and w_i are the weights that control the input of the corresponding i-th spot quality parameter into the overall quality value and are determined as described below. Assuming that the ratio variation coefficient in the k-th replicate, V_k, is proportional to the natural logarithm of Q_k

$$V_k \sim -Ln\left[\min_j\{\min_i\{q_{kji}^{w_i}\}\}\right] \qquad (2)$$

(the *Ln* transform is the most "natural" way to convert [0;1] scale of Q_k into [0;∞) scale of V_k) and including a proportionality constant V for the exponential transform of Eq. (2), Eq. (1) can be extended to

$$Q_k = \min_j\{\min_i\{q_{kji}^{w_i}\}\} = \exp(-V_k / V)\,. \qquad (3)$$

Where a constant coefficient V is the user-defined characteristic ratio variation coefficient. The weights w_i can be estimated from the best fit of the experimental quality values Q_k to the exponentially transformed ratio variation coefficient V_k [3]**.** The characteristic ratio variation coefficient, V, can be defined through two input parameters *Ratio CV Limit* and *Quality Limit* as *V = -Ratio CV Limit/ln(Quality Limit)*. These parameters should be defined by a user. Based on our tests we suggest to select initially *Ratio CV Limit* = 1 and *Quality Limit* = 0.1 before launching the procedure *Fit Limits* and then to set up them precisely after the procedure *Fit Limits* has been performed. *Ratio CV Limit* = 1 means that the standard deviation of two or more replicate ratios equals the average of the same ratios. *Quality Limit* = 0.1 is set up close to a range of a statistical error in this method, which is typically between 0.05 and 0.1. However, depending on the quality of a particular microarray the estimate *Quality Limit* might be lower and varied in the range [0;0.1]. *Ratio CV Limit* and *Quality Limit* should be set in Block b, where the initialization of the parameter

limits is performed. The quality parameter weights are then fitted in Block c *Fit Limits* using the abovementioned algorithms. Dots in the plot *Quality vs Ratio CV* are then regrouped as shown in Figure S1 D, so that the experimental quality dots are aligned along the user-defined quality (green) curve. Furthermore, the obtained weights are transformed into the admissible parameter limits taking into account the selected *Quality Limit*. These limits are calculated such that if a certain quality characteristic exceeds these limits, the corresponding (scaled) quality parameter will become lower than the selected *Quality Limit*. In Block d selecting a proper value of the *Quality Limit,* so to obtain on average the wished *Ratio CV Limit,* should be done. A user-decision for selecting "bad" spots has to be made from the desired value of the *Ratio CV*. For example, if one accepts on average the *Ratio CV* = 0.2, it means that errors are on average 20%, then the corresponding value of the *Quality Limit* from the green exponential line is calculated. Let assume it is 0.2 – then one should set-up the *Quality Limit* to 0.4 and features having quality values below 0.2 will be marked as "bad". Practically, the *Ratio CV limit* should be selected in the range [0.05;0.2] depending on the quality of a microarray. Generally a high value, close to 0.2, should be selected for a microarray of low quality – whereas 0.05 might be a good threshold for a microarray of high quality.

Frequently, the procedure of *Fit limits* over- or under-estimate the actual limits of the quality parameters. This is because different quality parameters are not totally independent leading to over-determined tasks. In practice, only a sub-set of all 10 parameters might be needed to describe adequately experimental variation in the microarray experiment. Therefore, we suggest to carefully identify the sub-set of the relevant quality parameters and to control their distributions to adjust their limits. Few typical examples of such limit adjustments for the parameters *Determination*,

CVRatio, and *Signal* for a selected microarray are shown in Figure S1 A-C. In this example**,** the left limit of the parameter *Determination* is underestimated, the right limit of the parameter *CVRatio* is overestimated, the left limit of the parameter *Signal* is underestimated. After manual adjustments of the corresponding limits the thresholds for the parameter distributions are corrected (right group of plots, Figure S1A-C) and the quality plot is recalculated.

The next step of the spot quality analysis is indeed rarely needed. It is applied if some spots that visually qualified as “bad” spots might still have overall quality value higher than the selected threshold. This is due to a technical limitation of the algorithms realized in MAIA, which might not be sensitive enough to classify some individual features using formulated filtering conditions. In this case a user must carefully check the image using the tools of *Manual Spot Characterization* (Block iv, Figure 1B, Manuscript).

Spot filtering in GP

Image analysis using the software GenePix Pro (GP) provides a powerful automatic tool for gridding, quantification, batch analysis that makes it easy following the analysis pipeline in the GP even for non-experienced users. However, the procedure of feature filtering is complicated and, thus, more intuitive than systematic. Although many publications devoted to microarray analysis using GP **–** majority of these studies applies default settings of the GP parameters and parameter limits for filtering features. Herewith, we try to systematize the GP filtering: i) to test various sets of the GP parameters and their cutoff values to define an optimal parametric set yielding a maximum of informative spots and ii) to make the results of the GP filtering comparable to the filtered datasets yielded by the image analysis in MAIA.

GP provides 56 parameters which can be separated into nine groups, representing logically-formalized properties of a spot in microarray. These groups were termed *Annotation, Geometry, Foreground, Background, Pixels, Intensity, Errors, Ratios, Saturation,* Table S1. Alternatively, we can reduce the total number of the GP parameters by performing hierarchical clustering of the parameters using the Pearson centred metrics for a typical microarray selected from our study. We omit seven spot-annotation parameters, *Block, Column, Row, Name, ID, X, Y*. The parameter *Flags* will be used in the follow-up analysis to filter not-present and not-found spots. Therefore, a set of parameters is reduced down to 46. The results of hierarchical clustering are shown in Figure S2 and the obtained clusters of the selected parameters at the correlation level 0.8 are listed in Table S2. We detected 13 clusters of GP parameters at the correlation level of 0.8. The parameters of the property *Errors* clustered into five clusters, numbered as 1-3, 8, 13, where the clusters 8 and 13 are shared by parameters of the properties *Foreground*, *Background*, and *Intensity*. The representatives of property *Background* appeared in clusters 4, 6, and 8, showing relation of the background intensities (in red and green channels) and errors. Parameters of the properties *Geometry*, *Saturation*, and *Ratios* were found present in distinct clusters. Parameters of *Foreground* and *Intensity* form the same cluster as well as parameters of *Errors* related to the signal intensities. The parameter *F Pixels* is correlated with the parameter *Dia*, and the parameter *B Pixels* forms independent cluster. Further, by combining the proper parameter representatives of each correlation group and logically-formalized property we reduce a full set of GP parameters to a limited and optimal set of the most representative parameters that is used further for automatic advanced filtering in GP.

The parameters of the property *Annotation* correspond to a user-specific filtering. Therefore, we omit them just leaving the parameter *Flags* that helps us to avoid situations where the not-found features are detected. Both parameters of the property *Geometry,* i.e. – *Dia* and *Circularity,* are important and sensitive to a change in the feature distributions, and, moreover, are not linearly correlated with other parameters. *Dia* and *Circularity* are selected for automatic filtering. Majority of parameters corresponding to the groups *Foreground* and *Background* are highly correlated and thus are not unique. We have selected the parameters *F635 Median* - *B635*, *F532 Median* - *B532* and % > *B635+2SD*, *% > B532+2SD* as main characteristics of *Foreground* and *Background* properties, respectively. For the same reason we omit estimations of the property *Pixels* – since *F Pixels* is in a high correlation with the parameter *Dia* and *B Pixels* is in a correlation with the property *Background*. The parameters of the property *Intensity* are in high correlation with the parameters of the property *Foreground* forming the biggest cluster in the dendrogram, Figure S2. As representatives of this group we selected two parameters *SNR 532*, *SNR 635*, that are also often used in other microarray studies. Investigation of the parameters of the group *Errors* has shown that many parameters of this property are not linearly correlated and are not informative or sensitive in our microarray series. In particular, this concerns the parameters *Ratios SD (635/532)*, *F532 CV*, *F635 CV*. In our study the parameter *Rgn R2 (635/532)* has been found most sensitive to the investigated features distributions and, therefore, it represents the property *Errors*. The estimations of the *Ratios* form one well-defined correlation cluster, the second largest cluster in the dendrogram. We do not use the parameters of the property *Ratios* for filtering, except the *Rgn Ratio* – that must be positive. The *Log Ratio* of the intensities in the Cy5 to Cy 3 channels is used in the further microarray analysis. Both parameters *F635*

% Sat., *F532 % Sat* from the property *Saturation* are linearly independent regarding other parameters and are chosen for automatic filtering. Finally, the parameters *Flags*, *Dia*, *Circularity*, *F635 Median - B635*, *F53 Median - B532, % > B635+2SD*, *% > B532+2SD*, *SNR 532*, *SNR 635*, *Rgn R2 (635/532)*, *Rgn Ratio (635/532)*, *F635 % Sat.*, *F532 % Sat.* have been selected for automatic filtering in GP.

The distributions of the selected GP parameters, for a typical microarray from this study, were investigated in order to define three groups of filtering conditions for detecting "good" spots. Based on approximation of 1 STD, 2 STD, and 3 STD borders of the tails in the parameter distributions, so-called *weak*, *medium* and *stringent/strong* filtering conditions were synthetically formulated. Summary of these constrains is shown in Table 1, Manuscript.

Other academic freeware with advanced features for image analysis

The module AMIA was developed as a toolbox for MATLAB that provides single-channel image analysis and only a set of diagnostic statistics to evaluate the data quality [4]. The image analysis package Matarray [5] is a MATLAB tool that performs spot quality analysis based on five quality characteristics: size of spot, signal to noise ratio, two measures for local background variability, and saturation. A draw-back of this approach is that the weight factors of the quality parameters are not taken in account in the composite quality score. The MASQOT-GUI utilizes two-channel microarray image analysis pipeline with an advanced automated multivariate quality control assessment but it hosts a set of independent applications for gridding, segmentation, quantification, quality assessment and data visualisation [6]. TIGR Spotfinder is an multichannel image processing program that provides basic spot quality control in terms of spot flags, scores, and p-values calculated for the spot area

(in pixels), spot perimeter, signal-to-noise ratio, distributions of the spot and background pixels [7]

References

1. Yang YH, Buckley MJ, Dudoit S, Speed TP: **Comparision of methods for image analysis on cDNA microarray data** *Journal of Computational and Graphical Statistics* 2002, **11**(1):1-29.
2. Novikov E, Barillot E: **A noise-resistant algorithm for grid finding in microarray image analysis**. *Machine Vision and Applications* 2006, **17**(5):337-345.
3. Novikov E, Barillot E: **An algorithm for automatic evaluation of the spot quality in two-color DNA microarray experiments**. *BMC Bioinformatics* 2005, **6**:293.
4. White AM, Daly DS, Willse AR, Protic M, Chandler DP: **Automated Microarray Image Analysis Toolbox for MATLAB**. *Bioinformatics* 2005, **21**(17):3578-3579.
5. Wang X, Ghosh S, Guo SW: **Quantitative quality control in microarray image processing and data acquisition**. *Nucleic Acids Res* 2001, **29**(15):E75-75.
6. Bylesjo M, Sjodin A, Eriksson D, Antti H, Moritz T, Jansson S, Trygg J: **MASQOT-GUI: spot quality assessment for the two-channel microarray platform**. *Bioinformatics* 2006, **22**(20):2554-2555.
7. Saeed AI, Sharov V, White J, Li J, Liang W, Bhagabati N, Braisted J, Klapa M, Currier T, Thiagarajan M *et al*: **TM4: a free, open-source system for**

microarray data management and analysis. *Biotechniques* 2003, **34**(2):374-378.

Figures

Figure S1 - Example of the spot quality analysis in MAIA using the parameter distribution plots (A-C) and the procedure of fit limits (D).

A

Parameter: *Determination*

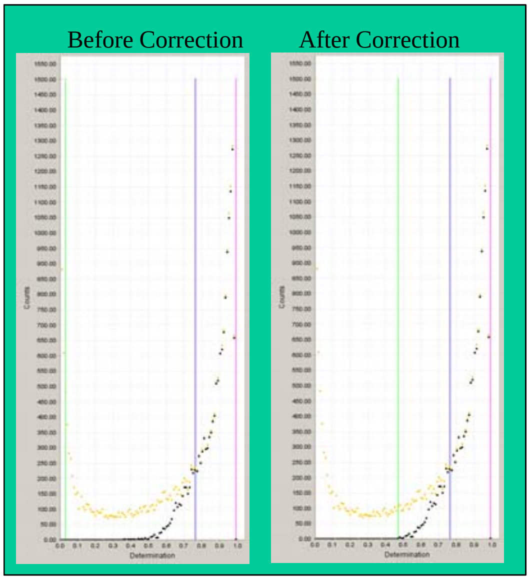

B

Parameter: *CVRatio*

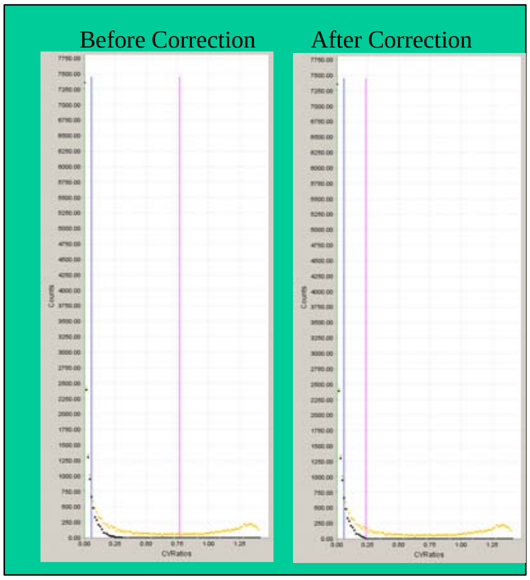

C

Parameter: *Signal*

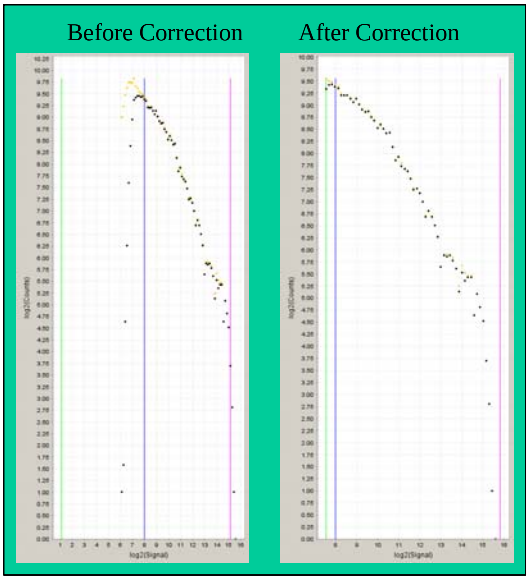

D

Before Fit Limits

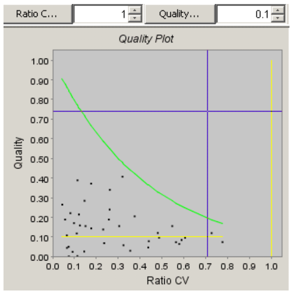

After Fit Limits

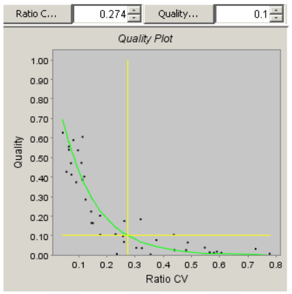

(A)-(C) Distribution plots of the quality parameters *Determination*, *CVRatio*, and *Signal*: examples of adjustments for the parameter limits for a selected microarray. “Good”- and “bad”-quality data are coloured in yellow and black accordingly. In the distribution plots the vertical lines indicate the parameter limits (green and pink) and the mean (blue). (D) *Quality vs Ratio CV* plot: results of the typical fit limits.

Figure S2 - Dendrogram of the 46 GP parameters resulting from the hierarchical clustering with the Pearson centered metrics applied to a typical microarray.

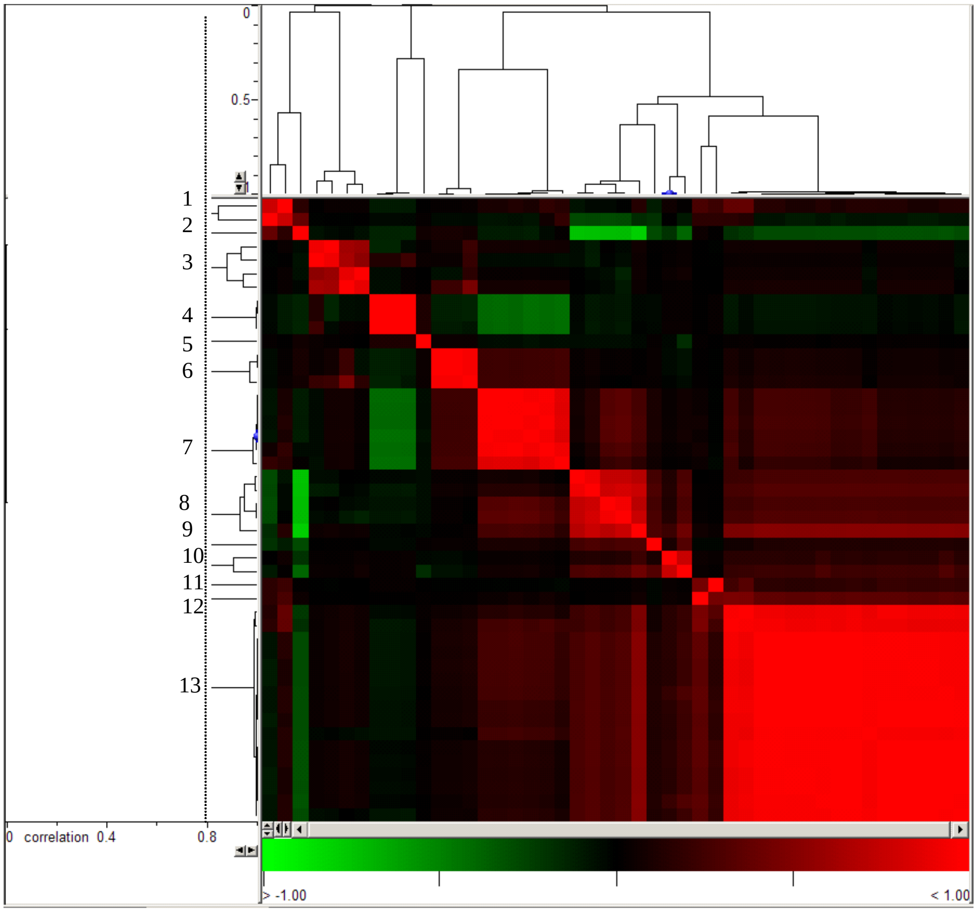

Two-dimensional dendrogram of the 46 GP parameters obtained by means of the hierarchical clustering using the Pearson-centred metrics. Numbering from 1 to 13 indicates the ordering of clusters of the GP parameters in Table S2.

Tables

Table S1 - The output GP parameters sorted in nine groups representing logically-formalized properties of a spot in a microarray.

Property	Parameter	Selected for analysis
Annotation	*Block, Column, Row, Name, ID, X, Y, Flags*	*Flags*
Geometry	*Dia., Circularity*	*Dia., Circularity*
Foreground	*F635 Median, F635 Mean, F532 Median, F532 Mean, F532 CV, F635 Median - B635, F532 Median - B532, F635 Mean - B635, F532 Mean - B532,*	*F635 Median - B635, F532 Median - B532*
Background	*B635, B635 Median, B635 Mean, % > B635+1SD, % > B635+2SD, B532, B532 Median, B532 Mean, % > B532+1SD, % > B532+2SD*	*% > B635+2SD, % > B532+2SD*
Pixels	*F Pixels, B Pixels*	–
Intensity	*Sum of Medians (635/532), Sum of Means (635/532), F532 Total Intensity, F635 Total Intensity, SNR 532, SNR 635*	*SNR 532, SNR 635*
Errors	*Ratios SD (635/532), Rgn R2 (635/532), F635 SD, F635 CV, F532 SD, B635 SD, B635 CV, B532 SD, B532 CV*	*Rgn R2 (635/532)*
Ratios	*Ratio of Medians (635/532), Ratio of Means (635/532), Median of Ratios (635/532), Mean of Ratios (635/532), Rgn Ratio (635/532), Log Ratio (635/532)*	*Rgn Ratio (635/532), Log Ratio (635/532)*
Saturation	*F635 % Sat., F532 % Sat.*	*F635 % Sat., F532 % Sat.*

Table S2 - Main clusters of the 46 GP parameters resulting from the hierarchical clustering with the Pearson centered metrics applied to a typical microarray.

Cluster number	Parameters	Associated Property	Selected for analysis
1	*F532 CV, F635 CV*	*Errors*	–
2	*Ratios SD (635/532)*	*Errors*	–
3	*B532 CV, B635 CV, B532 SD, B635 SD*	*Errors*	–
4	*B532, B532 Mean, B532 Median*	*Background*	–
5	*B Pixels*	*Pixels*	–
6	*B635, B635 Mean, B635 Median*	*Background*	–
7	*Ratio of Medians (635/532), Ratio of Means (635/532), Median of Ratios (635/532), Mean of Ratios (635/532), Rgn Ratio (635/532), Log Ratio (635/532)*	*Ratios*	*Rgn Ratio (635/532), Log Ratio (635/532)*
8	*B635+1SD, % > B635+2SD, % > B532+1SD, % > B532+2SD, Rgn R2 (635/532)*	*Background, Errors*	*% > B635+2SD, % > B532+2SD, Rgn R2 (635/532)*
9	*Circularity*	*Geometry*	*Circularity*
10	*Dia, F Pixels*	*Geometry, Pixels*	*Dia*
11	*F635 % Sat*	*Saturation*	*F635 % Sat*
12	*F532 % Sat.*	*Saturation*	*F532 % Sat.*
13	*F635 Median, F635 Mean, F635 SD, F532 Median, F532 Mean, F532 SD, F635 Median - B635, F532 Median - B532, F635 Mean - B635, F532 Mean - B532, SNR 532, SNR 635, Sum of Medians (635/532), Sum of Means (635/532), F532 Total Intensity, F635 Total Intensity*	*Foreground, Intensity, Errors*	*F635 Median - B635, F532 Median - B532, SNR 532, SNR 635*

The clusters are detected at the correlation level of 0.8.
