# Supplementary material for: Advanced spot quality analysis in two-colour microarray experiments
Source: BMC Res Notes. 2008 Sep 17;1:80. doi: 10.1186/1756-0500-1-80 (PMC2556690; doi:10.1186/1756-0500-1-80)
Supplement: Additional file 2 — Methods. Microarray fabrication, preparation of spiked RNA samples, microarray experiments, establishment of MCF-7 cell lines conditionally expressing SNAI1, RT-PCR and quantitative RT-PCR (qRT-PCR), statistical analysis of microarray data, software. [file 1756-0500-1-80-S2.pdf]

Methods

This study considered two sets of microarray experiments: i) control microarrays and ii) whole-genome microarrays from the SNAI1 induction experiment.

Microarray fabrication

Sequences of 60-mer oligonucleotides were derived from those of 70-mer probes corresponding to the *Arabidopsis thaliana* spiking control set originally developed at the Institute for Genomic Research (TIGR, Rockville, MD, USA) [1]. Oligonucleotides were synthesized, 3'-end amino (C6)-modified and HPLC-purified by Eurogentec (Seraing, Belgium). Microarrays were manufactured by contact printing using a Microgrid II microarrayer equipped with 2500 split pins (Genomic solutions, Huntingdon, United Kingdom). Oligonucleotides were spotted in hexaplicate onto epoxide-coated glass slides (ArrayIt, Sunnyvale, CA, USA) at ten different concentrations ranging from 1.2 to 45 µM in microspotting plus solution (ArrayIt). The library was printed with two array patches per slide, each containing 840 spots. Two human housekeeping genes and one bacterial sequence were included as positive and negative controls, respectively. Spotting was performed at a constant temperature of 22 °C with 50% controlled humidity. Following arraying, the slides were dried overnight and were stored desiccated at room temperature.

Preparation of spiked RNA samples

Spike poly(A+) RNAs were synthesised from the TIGR *Arabidopsis thaliana* spiking control pSP64 poly(A) vector set (Promega, Madison, WI, USA). Plasmids were linearised by *EcoR*I digestion, the restriction site being positioned immediately after the poly(A) tail sequence. One µg of each linearised plasmid was used as template for

the *in vitro* synthesis of sense transcripts using the MEGAscript High Yield Transcription kit (Ambion). Following DNAse I treatment, the transcribed RNAs were purified by lithium chloride precipitation and resuspended in 10 mM Tris-HCl pH 7.5. The quality and quantity of the RNA samples were assessed with a RNA Labchip (Agilent Biotechnologies) and classical spectrophotometry. Two 100x RNA mixes were then prepared, each containing a full range of spike RNAs at concentration ranging from 1000 to 30 000 cpc. Transcript copy number calculations were made assuming that a cell contains 1 pg poly(A) RNA corresponding to an average of 360 000 transcripts, and that 0.3 ng spike transcript corresponds to 100 spike copies/cell. Care was taken to use DEPC-treated water containing 1 µg/µl *E. coli* tRNA (Roche Diagnostics) to prevent the loss of spike RNAs at low concentrations through adsorption on plastic surfaces.

Microarray experiments

Control microarrays. One µg of poly(A+) RNA extracted from MCF-7 cells was combined with 1x *A. thaliana* control mix reverse-transcribed using the Superscript II reverse transcriptase and oligo(dT)$_{12-18}$ primer (Invitrogen). cDNAs were labelled with either Cy3 or Cy5 NHS-ester dye (GE Healthcare). The hybridisation was carried out at 42 °C for 20 h in a Slidebooster 800 (Advalytix, Brunnthal, Germany) with a regular microagitation of the sample. The microarrays were printed onto SuperEpoxy slides (ArrayIt) with 4 subgrids of 14 x 15 spots. The spike RNAs were combined in staggered concentration ranging from 10 to 300 copies per cell (cpc) to yield theoretical signal ratios of 1:1, 3:1 or 1:3 (Table S1). Slides were scanned immediately after post-hybridisation washing using a GenePix 4000B microarray fluorescence reader (Molecular Devices, Sunnyvale, CA, USA) at a resolution of 10 µm (for a typical image of the control slide see Figure S1).

Whole-genome microarrays. One and half microgram of total RNA from non-induced and induced samples was amplified by *in vitro* transcription using the Amino Allyl MessageAmp II aRNA Amplification kit (Ambion). Briefly, RNA was reverse transcribed using a T7 Oligo(dT) primer to generate first strand cDNA, containing a T7 promoter sequence. After second-strand synthesis with DNA polymerase and RNase H, the cDNA was purified and transcription performed using amino allyl-labelled dUTPs to generate antisense RNA (aRNA). Following aRNA purification, the amino allyl UTP residues on the aRNA were coupled to either Cy3 or Cy5 dye (GE Healthcare). The labelled aRNA was then hybridized onto Human Operon version 2.0 cDNA microarrays prepared by the "University Medical Center of Utrecht" (UMCU) containing 25 392 spots [2]. After denaturation of labelled aRNAs at 95°C for 3 minutes, hybridizations were carried out at 42°C for 16 to 20 h in a Slidebooster 800 (Advalytix, Brunnthal, Germany). The slides were washed in 3 different pre-warmed washing buffers at 42°C for 5 minutes (wash solution 1 : 2x SSC, 0.1% SDS; wash solution 2 : 1x SSC; wash solution 3: 0.5x SSC) before drying by centrifugation at room temperature for 2 minutes at 500 x g. A series of nine microarrays were performed including 2 dye-swaps out of nine slides, and the arrays were scanned as described above. Microarray data and procedures were deposited in the ArrayExpress public repository (www.ebi.ac.uk/arrayexress), or can be downloaded from the web site http://www.bioinformatics.lu .

Establishment of MCF-7 cell lines conditionally expressing SNAI1

To generate a MCF7 human breast adenocarcinoma cell line that conditionally expresses the human *SNAI1* gene, we used the tetracycline transactivator tetOff system (Clontech). Human *SNAI1* gene fused to a VSV-derived tag was cloned into pUHD10-3 vector [3] to obtain pUHD 10-3-SNAI1-VSV. pUHD 10-3-SNAI1-VSV

and pUHD10-3 as a control were transfected into MCF7-tetOff cells using calcium phosphate method together with the hygromycin-selectable vector pTK-Hyg (Clontech). Hygromycin resistant clones were selected and *SNAI1* gene expression in cells after withdrawal of tetracycline was gauged by real-time PCR and by immunofluorescence using anti VSV-antibodies. Well-characterised changes of the expression program in these cells were monitored at 96 hours after SNAI1 induction by microarrays analysis, RT-PCR, real-time PCR and by immunoblotting with antibodies directed against EMT marker proteins.

RT–PCR and quantitative RT–PCR

Total RNA was extracted from non-induced or induced MCF7-SNAI1 cells using the RNA NOW^{TM} reagent (Ozyme, St Quentin Yvelines), following manufacturer's instructions. Reverse Transcription (RT) of equal amounts of total RNA (1.5 µg) from non-induced and induced cells were performed using SuperScript III (SCIII) (Invitrogen) according to the manufacturer's instructions to obtain cDNA. RT-PCR and quantitative RT-PCR (qRT-PCR) amplification were done using the specific sense and anti-sense primers listed in Table S2. In both methods, Glyceraldehyde-3-phosphate dehydrogenase (GAPDH) was used as an endogenous control gene. All amplifications yielded amplicons of 70 to 160 nucleotides length. Each RT-PCR reaction (25 µl) was carried out using GoTaq^{TM} (Promega, Madison, WI, USA) and 1/40e µl of cDNA from SCIII. Amplification was performed using the following PCR program: 95°C, 5 min, for 1 cycle followed by 30 cycles (95°C, 30 seconds; 58°C, 30 seconds, and 72°C, 30 seconds). Half of the PCR product was analyzed on a 2% agarose gel to determine the presence of amplification products of expected size. Quantitative real-time PCR assays were performed with 1/40e µl of cDNA from SCIII using Brillant® Sybr® Green I QRT-PCR master system mix following

manufacturer's instructions (Stratagene Corporation, La Jolla, CA, USA). Real-time PCR assays were done with on Stratagene Mx3005P QRT-PCR machine. Amplifications were carried out with 1 cycle at 95°C for 10 min, followed by 40 cycles (95°C, 30 seconds; 58°C, 1 min, and 72°C, 1 min). Dissociation curve analysis was performed to verify the presence of a single PCR product. The average threshold cycle of triplicate reactions was used for all subsequent calculations using the ΔCt method [4].

Statistical analysis of microarray data

Control microarrays. We analyzed the Log_2 ratios of raw and calibrated data resulted from the analysis of control microarrays. The means of the obtained ratios from the *down-* and *up-* features were compared with a priory expected ratios using the error equation (1).

$$\delta = \frac{\left| M - M^* \right|}{M^*} 100\% \qquad (1)$$

where *M* is the real or *a priori* expected Log_2 ratio and *M** is the obtained Log ratio. To calculate the significance of differences in the comparisons we applied paired t-tests for two samples, assuming equal variances.

The microarray data analysis pipeline followed the workflow presented in Figure 2, Manuscript. Here, we give details for each step of the data analysis pipeline. We used the Log_2 transformation of the ratios of medians of the Cy3 and Cy5 background subtracted signal intensities for each spot. Genes with many missing values, typically those that were not present in at least 80% of microarrays, were considered as unreliable and were filtered out from the dataset. The good-quality data were normalized using the intensity-dependent print-tip lowess method [5]. Pre-processing steps included procedures of dye-swap conversion, evaluation and correction of genes

with missing values, data centring and scaling, data visualisations (box plots, histograms of the Log_2 ratios, MA-plots). Missing-values approximation was done using the K-nearest neighbours method [6]. Differential analysis of genes from replicated microarrays was done using the Significance Analysis of Microarrays (SAM) method [7]. Differentially expressed genes at the false-discovery rate of about 5% were selected from the SAM analysis and submitted to further gene ontology (GO) analysis. Classification into GO functional groups and analysis of over-represented themes were carried out using the client-server program package GoMiner [8, 9]. The complete human transcriptome was used for calculation of the expected frequencies in the over-representation analysis. GO mining utilized the facilities of the GoMiner platform that linked the databases LocusLink, PubMed, MedMiner, GeneCards, the NCBI's Structure Database, BioCarta, KEGG. The Fisher exact F-test and the permutation schemes (1000 permutations) were used to identify the relative enrichment of significant functional categories. A GO category was considered as over-represented if the FDR score was below 0.3.

Software

The image analysis was performed using the software MAIA 2.7 (see http://bioinfo-out.curie.fr/projects/maia/ and [10]) and GenePix Pro 6.0 (Molecular Devices, Sunnyvale, CA, USA). Data analysis of the results obtained with MAIA and GenePix was done using the commercial software Acuity 4.0 (Molecular Devices, Sunnyvale, CA, USA). Identification of differentially expressed genes was carried out using the MS Excel macro add-ins SAM 2.23 [7]. Statistical analysis was done using the MS Excel ToolPack *Data Analysis*. GO analysis and calculation of enriched functional categories were carried out using the client-server program package GoMiner [8, 9].

References

1. Wang HY, Malek RL, Kwitek AE, Greene AS, Luu TV, Behbahani B, Frank B, Quackenbush J, Lee NH: **Assessing unmodified 70-mer oligonucleotide probe performance on glass-slide microarrays**. *Genome Biol* 2003, **4**(1):R5.
2. Roepman P, Wessels LF, Kettelarij N, Kemmeren P, Miles AJ, Lijnzaad P, Tilanus MG, Koole R, Hordijk GJ, van der Vliet PC *et al*: **An expression profile for diagnosis of lymph node metastases from primary head and neck squamous cell carcinomas**. *Nature genetics* 2005, **37**(2):182-186.
3. Gossen M, Bujard H: **Tight control of gene expression in mammalian cells by tetracycline-responsive promoters**. *Proc Natl Acad Sci U S A* 1992, **89**(12):5547-5551.
4. Livak KJ, Schmittgen TD: **Analysis of relative gene expression data using real-time quantitative PCR and the 2(-Delta Delta C(T)) Method**. *Methods* 2001, **25**(4):402-408.
5. Yang YH, Dudoit S, Luu P, Lin DM, Peng V, Ngai J, Speed TP: **Normalization for cDNA microarray data: a robust composite method addressing single and multiple slide systematic variation**. *Nucleic Acids Res* 2002, **30**(4):e15.
6. Troyanskaya O, Cantor M, Sherlock G, Brown P, Hastie T, Tibshirani R, Botstein D, Altman RB: **Missing value estimation methods for DNA microarrays**. *Bioinformatics* 2001, **17**(6):520-525.
7. Tusher VG, Tibshirani R, Chu G: **Significance analysis of microarrays applied to the ionizing radiation response**. *Proc Natl Acad Sci U S A* 2001, **98**(9):5116-5121.

8. Zeeberg BR, Feng W, Wang G, Wang MD, Fojo AT, Sunshine M, Narasimhan S, Kane DW, Reinhold WC, Lababidi S *et al*: **GoMiner: a resource for biological interpretation of genomic and proteomic data**. *Genome Biol* 2003, **4**(4):R28.

9. Zeeberg BR, Qin H, Narasimhan S, Sunshine M, Cao H, Kane DW, Reimers M, Stephens RM, Bryant D, Burt SK *et al*: **High-Throughput GoMiner, an 'industrial-strength' integrative gene ontology tool for interpretation of multiple-microarray experiments, with application to studies of Common Variable Immune Deficiency (CVID)**. *BMC Bioinformatics* 2005, **6**:168.

10. Novikov E, Barillot E: **Software package for automatic microarray image analysis (MAIA)**. *Bioinformatics* 2007, **23**(5):639-640.

Figures

Figure S1 - Image of a control microarray.

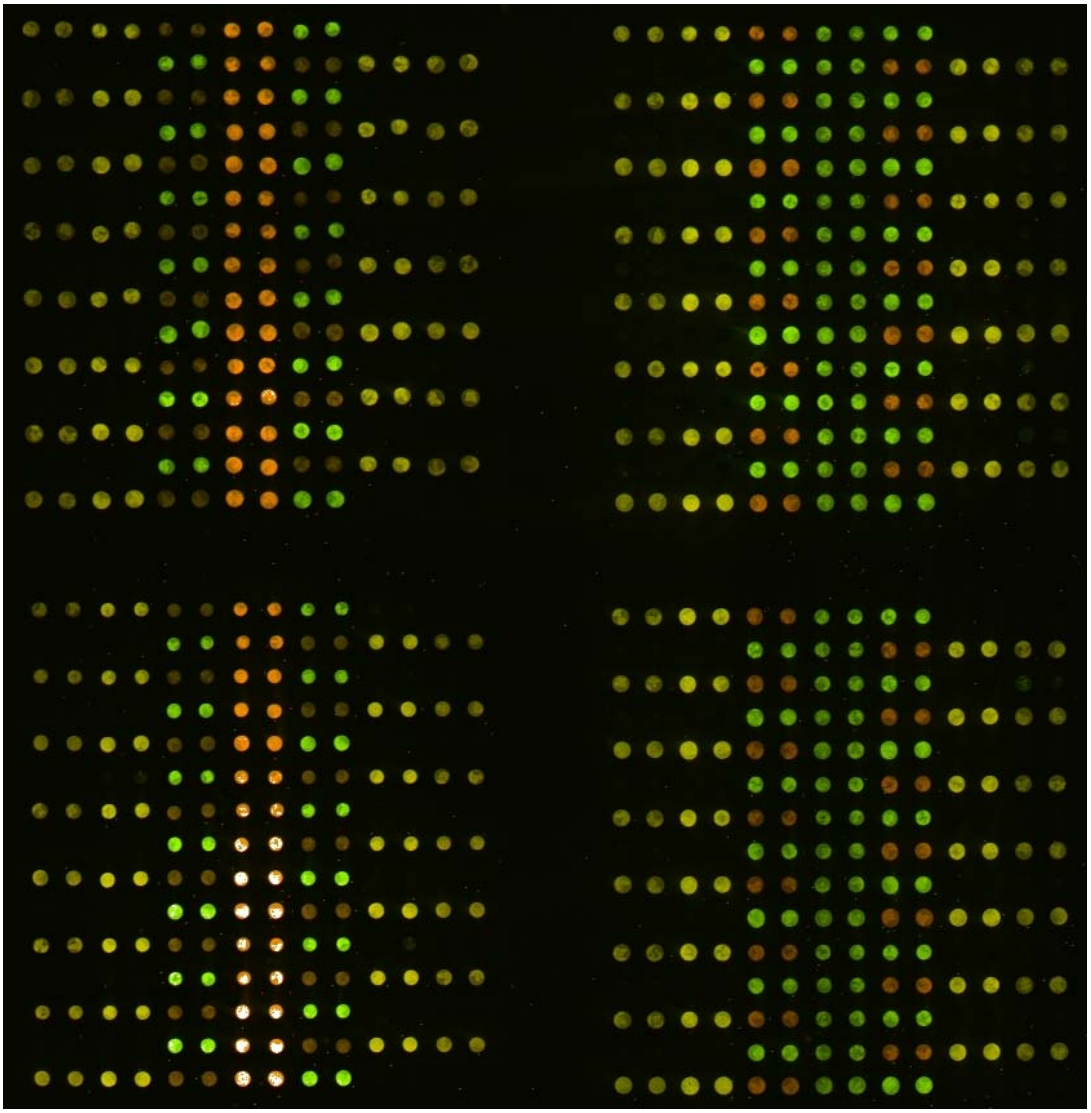

The control microarray is composed of 4 subgrids each containing 14 x15 spots, resulting in a total of 840 spots. The image was obtained by analysing a microarray following the hybridisation of fluorescently-labelled *Arabidopsis thaliana* spike RNAs combined in staggered concentration ranging from 10 to 300 copies per cell (cpc) to yield theoretical signal ratios of 1:1 (yellow spots), 3:1 (red spots) or 1:3

(green spots). Pseudocolours of spots are used in the usual way to describe the signal intensity ratios in the red and green channels.

Tables

Table S1 - The spike RNAs used for control microarrays.

Details for the spike RNAs used for control slides.

Spike RNA	Concentration (cpc)		Expression ratio (Cy5/Cy3)
	Cy5	Cy3	
CAB	50	50	1:1
LTP4	100	100	1:1
RCA	200	200	1:1
Rcbl	300	300	1:1
LTP6	30	10	3:1
RCP	120	40	3:1
NAC	450	150	3:1
XCP2	40	120	1:3
TIM	80	240	1:3
PRKase	100	300	1:3

Table S2 - Oligonucleotides.

Oligonucleotide primers used for RT-PCR and qRT-PCR amplification.

Type of assay	Short name	Forward primer	Reverse primer
RT-PCR	*KLF5*	ctgcctccagaggacctg	tcgtctatactttttatgctctggaat
RT-PCR	*TJP3*	atctggacggcggaagat	ggtgagggaggtctaggttgt
RT-PCR	*KRT12*	gcagattgacaatgcgagac	cagggccagttcattctcat
qRT–PCR	*BSPRY*	actcggagcccactactgac	cgtatgtcctctgtgcctga
RT-PCR	*CORO1A*	gggggatcactgtcctctc	aaacacgtggcggaactt
RT-PCR	*STAP2_HUMAN*	ggaaatgtggaaaggcttca	aggaagcagggtcaagtcg
RT-PCR	*PPP1R16A*	cctcccagtgttgtccttct	accccactcccaaggaac
qRT–PCR	*KRT18*	tgatgacaccaatatcacacga	ggcttgtaggccttttacttcc
RT-PCR	*STMN3*	gatggagctcagcaaggaga	cccttagcccgacatctct
qRT–PCR	*TRIB3*	gtcttcgctgaccgtgaga	cagtcagcacgcaggagtc
qRT–PCR	*CLDN3*	ctacgaccgcaaggactacg	gtggtggtgttggtggtg
RT-PCR	*TXNIP*	ttcgggttcagaagatcagg	ggatccaggaacgctaacat
RT-PCR	*MSX1*	ctcgtcaaagccgagagc	cggttcgtcttgtgtttgc
RT-PCR	*GULP1*	caagatttggaaaaccaactgag	gagggcgacttaggtgtcat
RT-PCR	*DUSP2*	ggccataggcttcattgact	gcatgaggtatgccagacag
RT-PCR	*ID3*	catctccaacgacaaaaggag	cttccggcaggagaggtt
RT-PCR	*THBD*	tacgggagacaacaacacca	aagtggaactcgcagaggaa
RT-PCR	*HS6ST2*	tgcgatcttctccaagattttc	cgatcacggcaaataggaag
RT-PCR	*TGFBI*	gacacctttgagacccttcg	cttcaagcatcgtgttgagc

RT-PCR	*S100A10*	gagttccctggatttttggaa	cactggtccaggtccttcat
RT-PCR	*SERPINH1*	gcgggctaagagtagaatcg	atggccaggaagtggtttg
qRT–PCR	*SNAI2*	tggttgcttcaaggacacat	gttgcagtgagggcaagaa
RT-PCR	*COL5A1*	cctggatgaggaggtgtttg	cggtggtccgagacaaag
RT-PCR	*ANXA2*	cccaagtggatcagcatcat	ccaacatgtcataagggctgt
