# Supplementary figures and images for: Advanced spot quality analysis in two-colour microarray experiments
Source: BMC Res Notes. 2008 Sep 17;1:80. doi: 10.1186/1756-0500-1-80 (PMC2556690; doi:10.1186/1756-0500-1-80)

A

MAIA

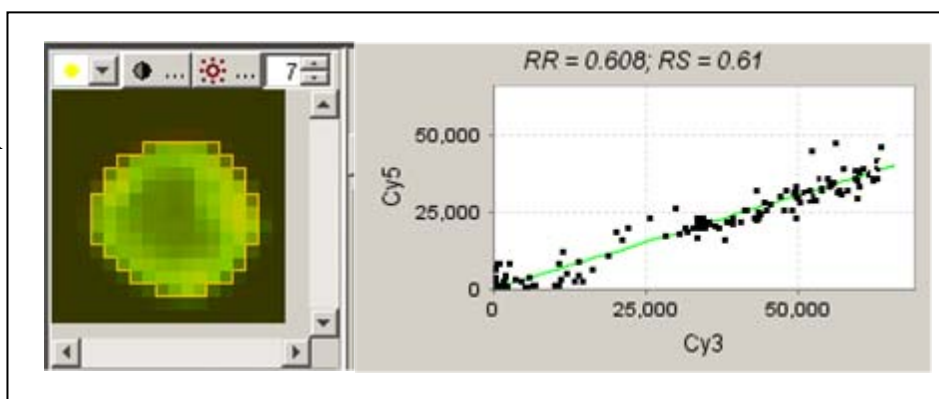

GP

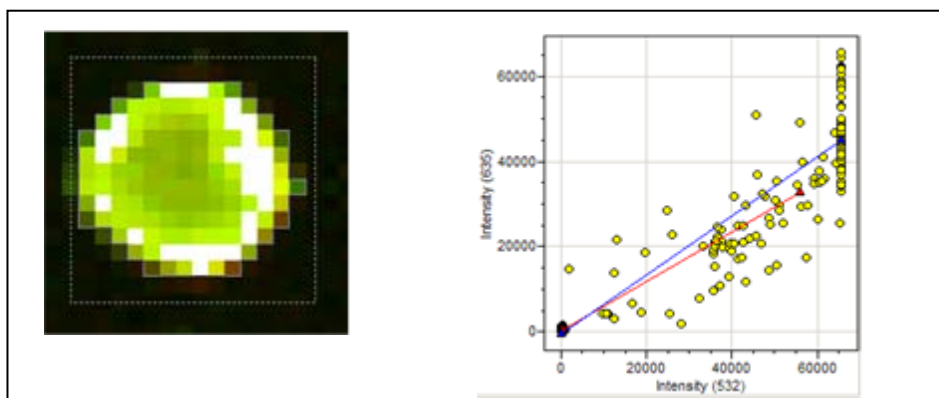

B

MAIA

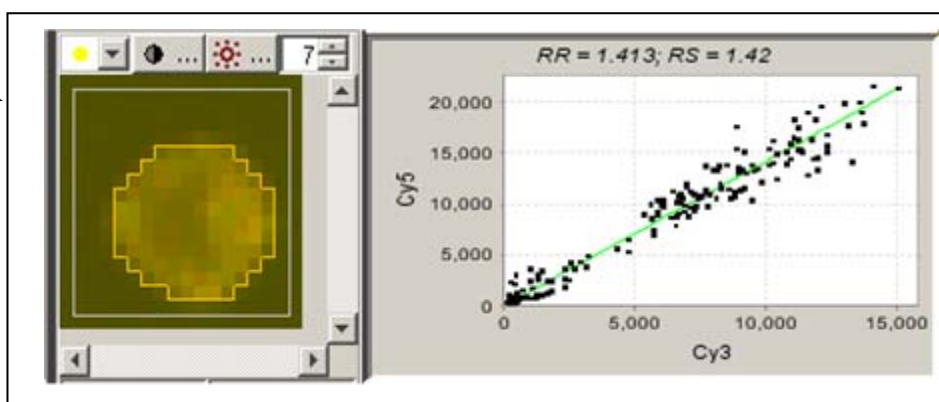

GP

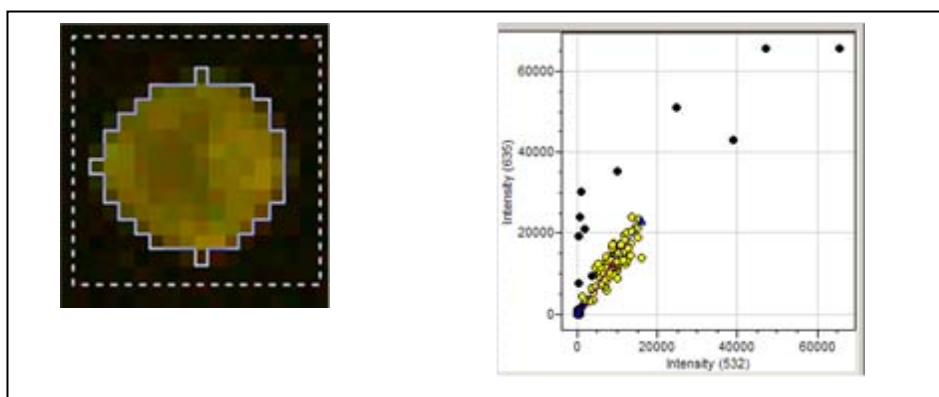

Supplement: Additional file 4 — Informative spots in the whole-genome microarray. Figure shows the images (left) and the Cy5/Cy3 scatter plots (right) of two typical informative spots that were removed by any filtering in GP and saved in MAIA during analysis of the whole-genome microarray data obtained in the SNAI1 induction experiment. (A) – A spot affected by a relatively high saturation effect, (B) – A spot affected by a high background intensity effect. [file 1756-0500-1-80-S4.pdf]
